# Effect of Potential-Determining Ions on Rheological Properties of Calcite Paste

**DOI:** 10.3390/ma18092020

**Published:** 2025-04-29

**Authors:** Jizhi Huang, Ruyu Li, Jiacheng Cai, Yu Wang, Jiansheng Chen, Hengbin Zheng

**Affiliations:** 1 College of Water Conservancy and Civil Engineering, South China Agricultural University, Guangzhou 510642, China; jizhihuang@scau.edu.cn (J.H.); 20233181008@stu.scau.edu.cn (R.L.); 202328310101@stu.scau.edu.cn (J.C.); 2 International School of Microelectronics, Dongguan University of Technology, Dongguan 523808, China; chnwangyu@dgut.edu.cn; 3 School of Civil Engineering, Hunan University of Technology, Zhuzhou 412007, China; jshchen2011@163.com; 4 Power China Zhongnan Engineering Corporation Limited, Changsha 410014, China

**Keywords:** calcite, zeta potential, rheology, electric double layer, potential-determining ions, DLVO theory

## Abstract

The mechanical properties of calcite suspension are predominantly affected by different ions dissolved in solution. In this work, natural and synthetic calcites were employed to investigate the influence of potential-determining ions (PDIs) (Ca^2+^, Mg^2+^, OH^−^, CO_3_^2−^ and SO_4_^2−^) on the zeta potential and rheological behavior of calcite paste. Electric double layer (EDL) models were proposed to further interpret the ionic adsorption mode and zeta potential evolution. Experimental results show that moderate addition of the positive PDIs Ca^2+^ and Mg^2+^ significantly increases the positive charge of calcite and enhances paste flow. Calcite exhibits higher zeta potential in Ca(NO_3_)_2_ but lower viscosity in Mg(NO_3_)_2_, which is attributed to the different affinity of Ca^2+^ and Mg^2+^ for the calcite surface. As for the negative PDIs OH^−^, CO_3_^2−^ and SO_4_^2−^, they make the calcite negatively charged with the order of $\left|\xi_{Na_{2}CO_{3}}\right|>\left|\xi_{Na_{2}SO_{4}}\right|>\left|\xi_{NaOH}\right|$. The negatively charged calcite paste exhibits much higher viscosity, which is against the conventional DLVO (Derjaguin–Landau–Verwey–Overbeek) theory. Lattice site screening and specific attraction induced by negative PDIs may be the reason for the phenomenon. This work provides a comprehensive understanding on the correlation between ionic adsorption, surface charge and particle interactions. These theories are enlightening for calcite application in many areas such as paper manufacturing, wall coating and heritage conservation.

## 1. Introduction

Calcite, one of the most widely distributed minerals in the world, plays a significant role in many environmental and geochemical processes such as CO_2_ sequestration and the storage of oil and gas [1,2,3,4]. Calcite minerals are also widely used in many fields, including paper and plastic filling [5,6], medicine production [7,8] and the construction industry [9,10,11,12,13]. Correlated to the application of calcite paste, its macroscopic behaviors such as fluidity and viscosity mainly depend on the interaction forces between calcite grains. According to the DLVO (Derjaguin–Landau–Verwey–Overbeek) theory [14,15], the interparticle force is dominated by the competing effect of electric double layer (EDL) repulsion and van der Waals attraction. The adsorption of different ions, mainly potential-determining ions (PDIs), can modify the surface charge of calcite, which in turn affects the EDL repulsion and the suspension flow.

Currently, it is widely accepted that Ca^2+^, Mg^2+^ and CO_3_^2−^, i.e., the main composition of carbonates, are the PDIs for calcite [16,17,18,19,20,21,22,23,24]. Other investigations believe that H^+^, OH^−^ and SO_4_^2−^ are also PDIs due to their good affinity to calcite surfaces [25]. As has been reported [26,27,28], the variation of pH can change the surface charge of calcite by protonation and deprotonation reactions. As for SO_4_^2−^, decreasing zeta potential and polarity changes were observed with increasing SO_4_^2−^ concentration [29,30,31]. The adsorption of PDIs can change the surface charge of calcite and hence the interparticle force and suspension flow. However, due to the complex interactions between calcite and different PDI species, the macroscopic behavior of calcite suspensions possibly disobeys the conventional DLVO model [3,8,32]. In recent studies, repulsive hydration force has been proposed as an explanation, and the short-distance repulsion was attributed to the compression and dehydration process of counterions adjacent to the calcite surface [33,34,35]. Other investigations reported short-distance attraction between cleaved calcite surfaces, and this was ascribed to ion–ion correlated force [36]. Due to the abnormal characteristics of calcite, the correlation between PDI adsorption, zeta potential and calcite suspension rheology remains unclear.

In order to reveal the effect of different ions on zeta potential of calcite and the interparticle forces in calcite suspension, in this work, two calcites from natural and synthetic sources were employed, and a series of model solutions containing different suggested PDIs or indifferent ions with varied concentration were prepared to test the zeta potential of natural and synthetic calcite. EDL models were also proposed to further interpret the ionic adsorption mode and zeta potential evolution. Meanwhile, a rheology test was conducted on natural calcite paste to interpret the dominant interactions in dense calcite suspension. The results exhibit a comprehensive understanding on the correlation between ionic adsorption, zeta potential and calcite paste rheology; they also present a vital insight for calcite application in many fields using surface charge control.

## 2. Materials and Methods

### 2.1. Materials and Samples

**Figure 1:**
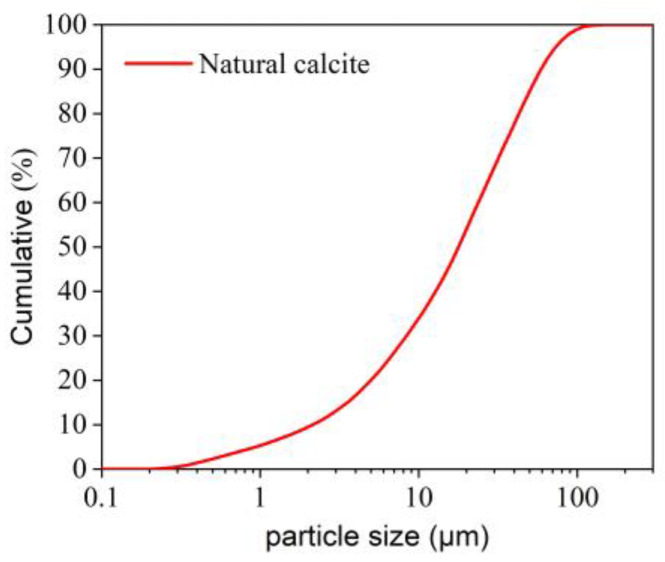
Particle size distribution of natural calcite.

In this research, two calcites from natural and synthetic sources were used for experiments. The synthetic calcite was an analytical reagent of calcium carbonate (AR CaCO_3_), and the natural calcite was a commercial product from Yangzhou, China, with an average particle size of 5.7 μm; the particle size distribution of natural calcite is shown in Figure 1. The chemical compositions of the two calcites are listed in Table 1. The composition of the synthetic calcite is provided by the manufacturer (Tianjin Fuchen Chemical Reagent Factory, Tianjin, China), whereas the composition of natural calcite was determined by X-ray Fluorescence Spectrometer.

### 2.2. Zeta Potential Test and DLVO Theory

**Figure 2:**
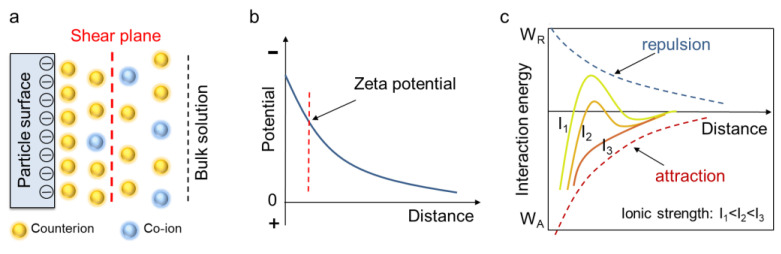
Zeta potential and DLVO theory. (**a**) Formation of electric double layer due to ionic adsorption. (**b**) Schematic illustration of zeta potential. (**c**) DLVO theory and the effect of ionic strength.

The surface of minerals usually carries a different charge in aqueous solution. Negatively charged surfaces, for instance, can adsorb cations (i.e., counterions) to balance the surface charge, which forms the electric double layer (EDL) (see Figure 2a). During the electrophoresis process, counterions adjacent to the mineral surface are firmly adsorbed and will move together with the particle, whereas the counterions at a greater distance from the mineral surface remain with the bulk solution. The sliding boundary between these counterion layers is called the shear plane, and the potential at the shear plane is the zeta potential, which can be determined by equipment (Figure 2b). Particles containing the same charge exhibit electric repulsion (or EDL repulsion) between each other, which helps to keep the suspension stable; on the other hand, there is also van der Waals attraction between particles, which leads to flocculation and accumulation. The competitive effect of electric repulsion and van der Waals attraction is described in the DLVO theory, which was proposed by Derjaguin, Landau, Verwey and Overbeek in 1948 [14,15]. The valance and concentration of ions (collectively named ionic strength) can change the zeta potential as well as the magnitude of the electric repulsion, thus altering the DLVO curve and the status of a mineral suspension. Zeta potential testing and DLVO theory provide an effective approach to investigate the rheological properties of different mineral suspensions, since the ionic adsorption can tune the particle interactions, thus enhancing or inhibiting suspension flow.

In order to reveal the effect of different ions (Na^+^, K^+^, Ca^2+^, Mg^2+^, OH^−^, CO_3_^2−^ and SO_4_^2−^) on the zeta potential and rheological behavior of calcite paste, a series of model solutions were prepared with the analytical reagents NaNO_3_, KNO_3_, Ca(NO_3_)_2_, Mg(NO_3_)_2_, NaOH, Na_2_CO_3_ and Na_2_SO_4_ in deionized water (DIW), and the concentration varied from 0.1 to 80 mM. In order to maintain the ionic strength at low concentrations, a background electrolyte of 10 mM NaNO_3_ was introduced in all tested solutions. It should be noted that the NaOH and Na_2_CO_3_ increase the alkalinity of the solution, so the pH was recorded for further analysis. All the solutions were freshly made to avoid the influence of atmospheric CO_2_.

Zeta potential tests were conducted with a NanoBrook Omni zeta potential analyzer (Brookhaven Instruments Corporation, Holtsville, NY, USA) based on a phase-analysis light-scattering method. The sample suspension was prepared by dispersing about 0.001 g calcite in 1.25 mL of model solution. In order to minimize the effect of particle settling, all samples were kept stationary for two minutes before measuring. Three replicated measurements with ten measuring cycles were conducted for each zeta potential test.

### 2.3. Rheology Test

**Figure 3:**
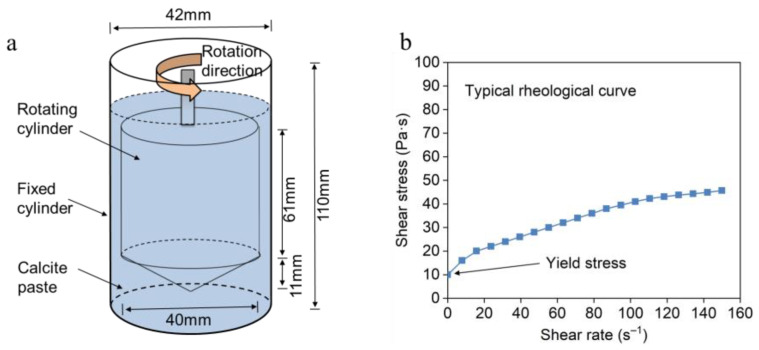
Schematic illustration of rheology test (arrow indicates the direction of rotation). (**a**) Coaxial cylinder with 68.5 mL sample paste inside. (**b**) Typical rheological curves of calcite paste (slope of the curve corresponds to the paste viscosity).

The rheology test was conducted using a Brookfield R/S Plus Rheometer (Brookfield, Middleboro, MA, USA) with a coaxial cylinder of type CC40 at room temperature. The size of the coaxial cylinder is shown in Figure 3a. The calcite paste was prepared by mixing 200 g of natural calcite with 90 g model solution in a Hobart mixer, thus obtaining a dense calcite paste with a solid–liquid ratio of 0.45 by weight. Then, 68.5 mL of fresh mixed calcite paste was transferred into the cylinder and the rheological test was conducted immediately. The concentrations of model solutions for rheological experiments were in the gradient of 1 mM, 10 mM, 20 mM, 40 mM and 80 mM, with 10 mM background electrolyte NaNO_3_ included. Compared with the concentration of model solutions, the ionic increase induced by calcite dissolution is much smaller (0.15 mM), which has negligible effects on the rheological behavior of calcite paste. The rheological curves were obtained by increasing the shear rate from 0 to 150 s^−1^ in 90 s, and shear stress and viscosity were automatically recorded by the equipment.

## 3. Results and Analysis

### 3.1. Effect of Indifferent Ions

**Figure 4:**
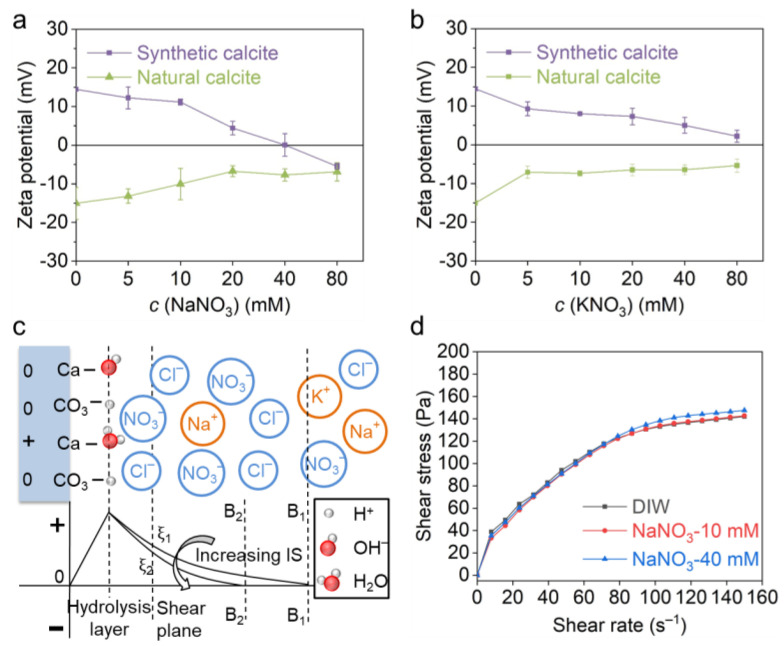
Zeta potential and rheological behavior of calcite paste with indifferent ions. The effect of (**a**) Na^+^ and (**b**) K^+^ on zeta potential of natural and synthetic calcite. (**c**) EDL structure of synthetic calcite in a solution of indifferent ions and the effect of ionic strength on zeta potential evolution. (**d**) The effect of NaNO_3_ concentration on rheological behavior of natural calcite paste.

Figure 4a,b exhibit the zeta potential evolution of two calcites in NaNO_3_ and KNO_3_ solutions. The result shows that the synthetic calcite is positively charged in pure water (+14.5 mV), whereas the natural calcite is negative (−15.0 mV). With an increase in electrolyte concentration, the zeta potential of both calcites decreases, and the gradient seems higher in the NaNO_3_ solution; typically, an isoelectric point (IEP) was observed for synthetic calcite when the NaNO_3_ exceeded 40 mM. This distinction between Na^+^ and K^+^ is probably due to their different affinities to the carbonate surface, which is characterized by the “Covalency Index (CI)” and “Ionicity Index (II)” by Marchuk et al. [37,38,39]. The zeta potential result indicates that Na^+^, K^+^ and NO^3−^ are not PDIs for carbonates, and they change the zeta potential simply by tuning the EDL thickness (Figure 4c). The IEP observed for the synthetic calcite in the NaNO_3_ solution is attributed to the overcompensation of counterions rather than specific adsorption.

The effect of the NaNO_3_ concentration on the rheological behavior of natural calcite is shown in Figure 4d. Similar rheological curves were obtained in 10 mM NaNO_3_ solution and deionized water (DIW). When NaNO_3_ increased from 10 mM to 40 mM, the shear stress increased slightly from 142.7 Pa to 147.5 Pa at a shear rate of 150 s^−1^. The rheology of this calcite paste indicates that the increasing indifferent ions have a very small effect on the interparticle force, which is in accordance with the zeta potential result.

### 3.2. Effect of Positive PDIs

**Figure 5:**
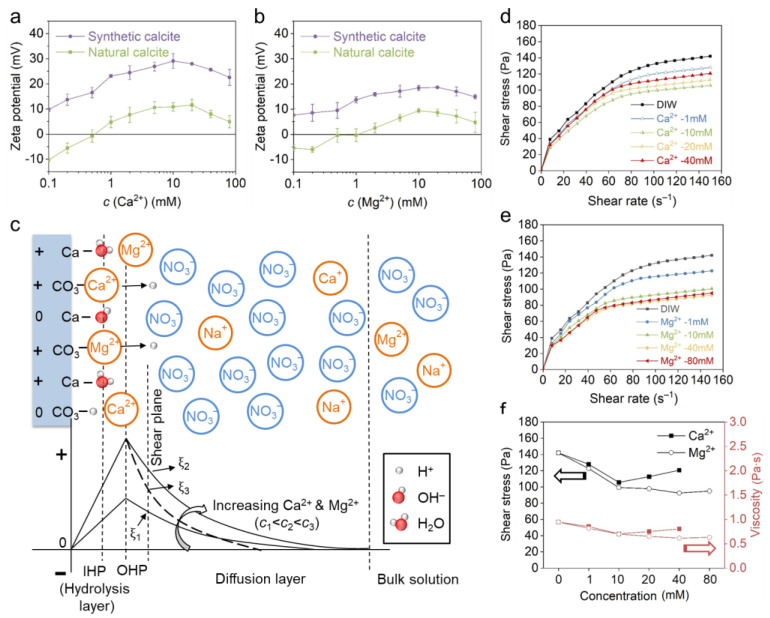
The effect of (**a**) Ca^2+^ and (**b**) Mg^2+^ on zeta potential of natural and synthetic calcite. (**c**) The EDL structure of synthetic calcite and zeta potential evolution in increasing PDI concentration. The effect of (**d**) Ca^2+^ and (**e**) Mg^2+^ on natural calcite paste rheology. (**f**) Comparison between the effect of Ca^2+^ and Mg^2+^ on the shear stress and viscosity at a shear rate of 150 s^−1^ (the black and red lines indicate the shear stress and viscosity, respectively).

The influence of the positive PDIs Ca^2+^ and Mg^2+^ on the zeta potential and rheological behavior of calcite paste was investigated with Ca(NO_3_)_2_ and Mg(NO_3_)_2_ solutions. Figure 5a,b show that both Ca^2+^ and Mg^2+^ can elevate the positive charge of calcites. For natural calcite, an isoelectric point (IEP) was observed at approximately 0.5 mM in both electrolytes, whereas the synthetic calcite was constantly positive over the entire concentration range. The maximum zeta potential was obtained at 10 mM or 20 mM, above which the zeta potential decreased again due to EDL compression. The zeta potential result confirmed that both Ca^2+^ and Mg^2+^ are PDIs for carbonates, and they are specifically adsorbed irrespective of the initial charge of different calcites. The configuration of the EDL was further illustrated in Figure 5c. According to previous reports [40,41,42], the specifically adsorbed Ca^2+^ and Mg^2+^ mainly distribute within the outer Helmholtz plane (OHP), which promotes the electric potential of the OHP and consequently the zeta potential at the shear plane. On the other hand, the negative counterions of NO_3_^−^ are arranged in the diffusion layer by electrostatic attraction to ensure the neutrality of the EDL. The peak zeta potential indicates the completion of monolayer adsorption [18,20], and excessive PDIs will decrease the zeta potential by reducing the Debye length. It is also observed in Figure 5a,b that the synthetic calcite gains a much higher zeta potential in Ca^2+^ than in the condition of Mg^2+^; this may result from a closer shear plane to calcite surface distance, since the Ca^2+^ is more strongly bound due to a higher covalent index (Ca^2+^ = 0.33, Mg^2+^ = 0.27) [37,38,39]. It is noted that natural calcite is constantly more negative than synthetic calcite, which is in accordance with previous reports [21,42,43,44,45,46,47], and this is attributed to the presence of impurities (see Table 1) [17,48]. In natural conditions, impurities such as environmental solutes, minerals and organic matters may co-precipitate with calcite [49,50], which leads to a different initial charge on carbonates and consequently a different zeta potential evolution in model solutions.

The rheological behavior of natural calcite paste with Ca(NO_3_)_2_ solution is shown in Figure 5d. With the increase in Ca^2+^ concentration, the rheological curve descends significantly and reaches a minimum at 10 mM. On further increasing the concentration to 20 mM, the shear stress rises again due to EDL compression. The effect of Ca^2+^ on calcite paste rheology is basically in accordance with the zeta potential result (see Figure 5a). Obviously, moderate Ca^2+^ adsorption promotes the surface charge and enhances the EDL repulsion. In comparison, calcite reaches the minimum shear stress at a much higher concentration (40 mM) in Mg^2+^. Furthermore, the shear stress and viscosity are always smaller in Mg^2+^ than Ca^2+^ (Figure 5f), even though a higher zeta potential was obtained in Ca^2+^ at the same concentration. The difference between Ca^2+^ and Mg^2+^ is also probably attributed to their affinity to the calcite surface, which will be discussed in Section 4.2.

### 3.3. Effect of Negative PDIs

#### 3.3.1. The Effect of OH^−^

**Figure 6:**
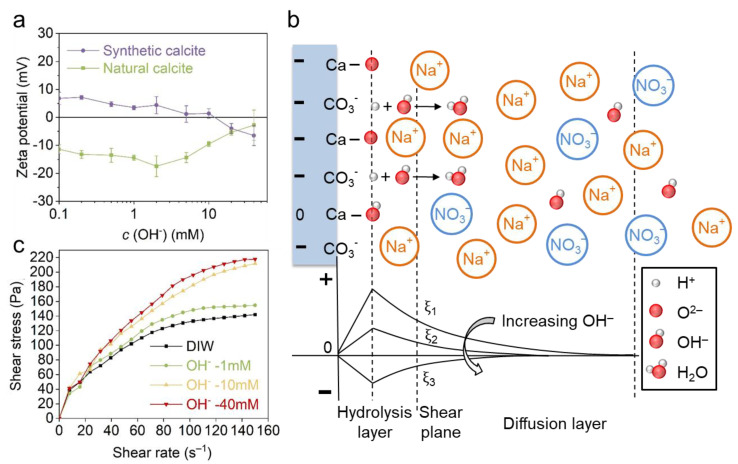
(**a**) The effect of OH^−^ on the zeta potential of natural and synthetic calcite. (**b**) The EDL structure of synthetic calcite and the zeta potential evolution in OH^−^. (**c**) The effect of OH^−^ on the rheological behavior of natural calcite paste.

Figure 6a shows that the zeta potential of synthetic calcite decreases monotonically with the increasing NaOH concentration, and the gradient is about −4.8 mV/decade. Particularly, an IEP was observed at 5–10 mM, which indicates the OH^−^ is also a PDI for calcite. The possible configuration of OH^−^ adsorption is further illustrated in Figure 6b. Different from Ca^2+^ and Mg^2+^ (also for CO_3_^2−^), the OH^−^ ions mainly change the calcite charge in the hydrolysis layer (i.e., the hydrated >CaOH and >CO_3_H sites, where “>” denotes chemical bond) by deprotonation [5,25,26,27,28]. As for natural calcite, the zeta potential firstly increases to a more negative state and then decreases again. The subsequent decrease may result from the enhanced adsorption of Na^+^ by impurities, which results in charge neutralization. A similar zeta potential trend was also reported in our previous investigation on quartz [38].

The rheological behavior of natural calcite with NaOH solutions was further investigated (Figure 6c). With the increase in OH^−^ concentration, a drastically increasing shear stress was observed on the rheological curves, even though the zeta potential varied within a very small range. Previous reports ascribed this phenomenon to ionic strength and electrostatic screening; however, in comparison to indifferent ions (see Figure 4 in Section 3.1), calcite paste exhibits much higher shear stress in OH^−^, even though ionic strengths are identical in the two electrolytes. The abnormal stiffening indicates a specific attraction between calcite particles which is beyond the DLVO correlated forces (EDL repulsion and van der Waals attraction), and this specific attraction leads to flocculation and hinders the paste flow.

#### 3.3.2. The Effect of CO_3_^2−^

**Figure 7:**
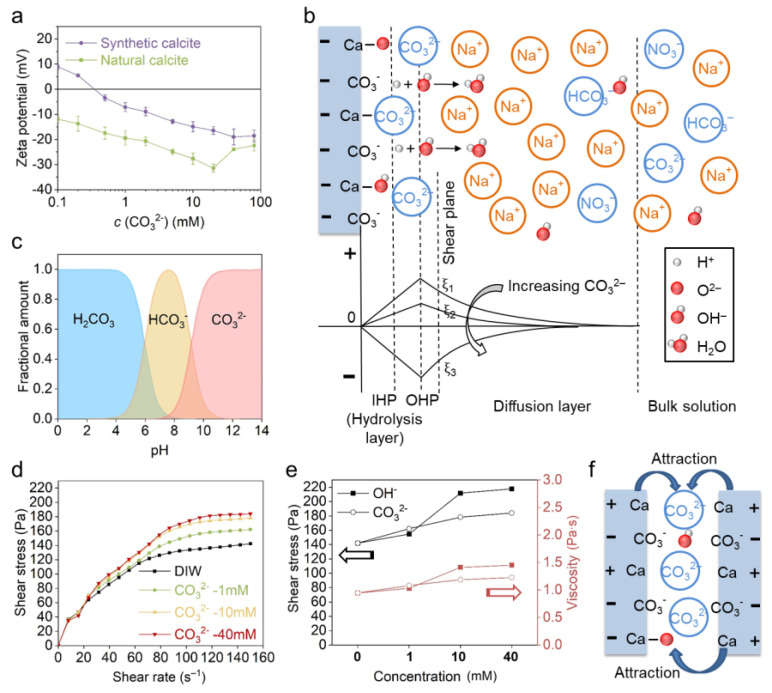
(**a**) The effect of Na_2_CO_3_ on the zeta potential of natural and synthetic calcite. (**b**) The EDL structure of synthetic calcite in Na_2_CO_3_ solution and the evolution of zeta potential. (**c**) The fractional amount of carbonate species as a function of pH, adapted from Mahrouqi et al. [29,42]. (**d**) The rheological behavior of natural calcite paste with Na_2_CO_3_ solutions. (**e**) Comparison between the effect of NaOH and Na_2_CO_3_ on the shear stress and viscosity at a shear rate of 150 s^−1^ (the black and red lines indicate the shear stress and viscosity, respectively). (**f**) The specific attraction between calcite particles induced by the negative intermediate Helmholtz layer.

The effect of CO_3_^2−^ was investigated with model solutions of Na_2_CO_3_ reagent. The zeta potential result (Figure 7a) shows that both calcites become more negatively charged with the increasing Na_2_CO_3_ concentration, and a linear regression with a gradient of −8.2 mV/decade can be fitted to both curves within 0.5–20 mM. When the concentration exceeded 20 mM (or 40 mM for synthetic calcite), the zeta potential decreased again due to EDL collapse and enhanced adsorption of positive Na^+^ ions. The mechanism of CO_3_^2−^ adsorption and zeta potential evolution was further illustrated in Figure 7b. Contrary to positive PDIs, the adsorption of CO_3_^2−^ generates a negatively charged surface, and the positive Na^+^ counterions are arranged in the diffusion layer to balance the negative charge. It is noted that in Na_2_CO_3_ solution, the evolution of calcite charge is correlated to pH and the ionic species in solution. Firstly, possible carbonate species like H_2_CO_3_, HCO_3_^−^ and CO_3_^2−^ in Na_2_CO_3_ solution are highly dependent on pH (Figure 7c). With the increase in Na_2_CO_3_ concentration and consequently the pH (Table 2), more monovalent HCO_3_^−^ ions transform to divalent CO_3_^2−^ and increase the negative charge. Secondly, the alkaline solution also leads to a deprotonation reaction on the calcite surface and enhances the negative charge [5,25,26,27,28].

The higher CO_3_^2−^ concentration also leads to higher shear stress and viscosity (Figure 7d), which is similar to the effect seen in NaOH solution. However, the CO_3_^2−^ seems less effective than OH^−^, because both shear stress and viscosity are lower in CO_3_^2−^ than OH^−^ at 10 mM and 40 mM conditions, as shown in Figure 7e. The pH results in Table 2 indicate that the distinct rheological behavior of calcite in OH^−^ and CO_3_^2−^ results from the different pH, the higher pH (i.e., OH^−^ concentration) creating the greater attractive force between calcite particles which hinders the suspension flow. From the rheology test, we also see a non-DLVO behavior in CO_3_^2−^ since the calcite is highly negatively charged, which should generate repulsion and improve the paste flow. Herein, we proposed a specific attraction between calcite particles, and the mechanism is illustrated in Figure 7f. In the alkaline Na_2_CO_3_ solution, the intermediate negative Helmholtz layer, consisting of the negative PDI CO_3_^2−^ and deprotonated sites >CaO^−^ can attract neighboring particles, which in turn inhibits the paste flow.

#### 3.3.3. The Effect of SO_4_^2−^

**Figure 8:**
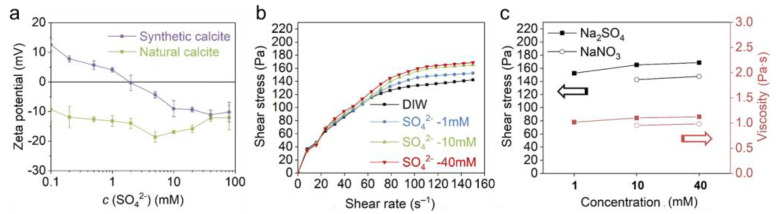
(**a**) The effect of SO_4_^2−^ on the zeta potential of natural and synthetic calcite. (**b**) The effect of SO_4_^2−^ on the rheological behavior of natural calcite paste. (**c**) Comparison between the effect of Na_2_SO_4_ and NaNO_3_ on calcite paste rheology at 150 s^−1^ (the black and red lines indicate the shear stress and viscosity, respectively).

The effect of SO_4_^2−^ was investigated with Na_2_SO_4_ solutions. Herein, the effect of pH can be ignored since the Na_2_SO_4_ solution is neutral. Figure 8a shows that the zeta potential of synthetic calcite constantly becomes more negative when Na_2_SO_4_ concentration increased to 40 mM, and the gradient is about −9.3 mV/decade. In addition, an IEP of 2 mM was also observed on the zeta potential curve. In contrast, the zeta potential of natural calcite is relatively moderate with an inflection point at 5 mM. The different zeta potential between natural and synthetic calcite is also attributed to the impurities, which leads to a different initial charge and consequently the zeta potential evolution. In addition, no IEP was observed on natural calcite since it is constantly negatively charged over the entire SO_4_^2−^ range.

With respect to the calcite paste rheology (Figure 8b), the increasing SO_4_^2−^ concentration also leads to higher shear stress. In comparison with OH^−^ and CO_3_^2−^, the effect of SO_4_^2−^ is less pronounced, but it is more effective than indifferent ions when the ionic strength is taken into consideration (Figure 8c). The stiffening of the calcite suspension indicates that, similar to CO_3_^2−^, the SO_4_^2−^ is also specifically adsorbed in the Helmholtz layer, thus leading to a specific attraction between calcite particles. Our suggestion is supported by Pourchet et al. [10]: it was detected by atomic force microscopy (AFM) measurement that a small amount of sulfate adsorption significantly increased the attractive interaction between calcite particles. The mechanism of SO_4_^2−^ adsorption and particle attraction is referred to in Figure 7b,f.

## 4. Discussion

### 4.1. Zeta Potential and PDI Determination

In this work, the zeta potential of natural calcite is constantly more negative than synthetic calcite due to the presence of impurities, and similar results have also been reported by other researchers [17,48,49,50]. Given this, the identification of a PDI should not rely on the emergence of an IEP during the zeta potential test, because the IEP might not be encountered when the ionic polarity is the same as the initial charge of the calcite. For instance, the synthetic calcite exhibits no IEP throughout the entire Ca^2+^ and Mg^2+^ concentration range although they are both confirmed PDIs, and similarly, the natural calcite also has no IEP in OH^−^, CO_3_^2−^ or SO_4_^2−^ solutions. Herein, we proposed that the solubility of precipitates is feasible to define a PDI, or at least sequence the affinity of different ions to the calcite surface. For example, in this work, the solubility of anionic compounds follows the order of $S_{CaCO_{3}}<S_{CaSO_{4}}<S_{Ca{\left(OH\right)}_{2}}$, which is opposite to the maximum zeta potential of $\left|\xi_{CO_{3}^{2-}}\right|>\left|\xi_{SO_{4}^{2-}}\right|>\left|\xi_{OH^{-}}\right|$. Similarly, the solubility of $S_{CaCO_{3}}<S_{MgCO_{3}}$ is also opposite to the zeta potential of $\left|\xi_{Ca^{2+}}\right|>\left|\xi_{Mg^{2+}}\right|$. Our suggestion is supported by Fajans’ rule [51,52,53], which predicts that the ions that can form insoluble matter with the lattice ions of a matrix will be preferentially adsorbed. Furthermore, the rheology test in this research also indicates a feasible approach for PDI identification, since the calcite paste is insensitive to the concentration of indifferent ions.

### 4.2. The Non-DLVO Behavior of Calcite Paste

In this work, non-DLVO behavior was observed in negative PDI conditions, where negatively charged calcite paste exhibits higher shear stress and viscosity. A similar result was also reported in other publications: Pourchet et al. [10] detected by AFM measurement an increasing attractive force between calcite particles when SO_4_^2−^ was incorporated. Liberto et al. [8] also reported that NaOH completely screens the surface charge and leads to a more rigid calcite paste. In previous reports, the calcite stiffening was ascribed to ionic strength and electrostatic screening [8]. However, this cannot explain why the negative zeta potential generates attraction rather than repulsion. Moreover, it also cannot explain the distinct rheological behavior between OH^−^ and indifferent ions, i.e., both ions have a very small effect on zeta potential, but the OH^−^ significantly increased the viscosity of calcite paste whereas the indifferent ions have a negligible effect.

**Figure 9:**
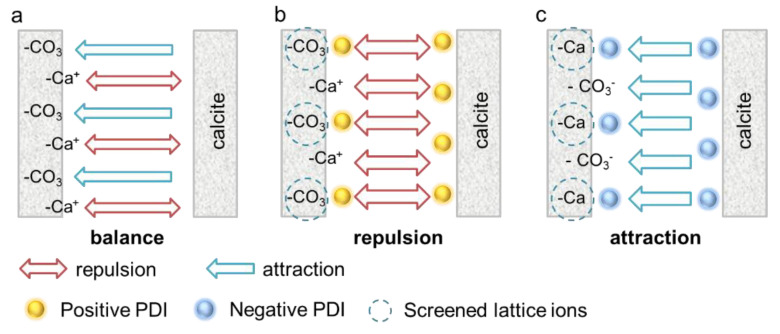
Specific interaction between calcite surfaces with the addition of different PDIs. (**a**) Calcite without PDI addition. (**b**) Positive PDIs screened the negative >CO_3_^2−^ sites, thus enhanced EDL repulsion. (**c**) Negative PDIs screened the positive >Ca^2+^ sites and induced a specific attraction.

It is proposed here that the non-DLVO behavior in negative PDIs is dominated by a specific attraction beyond the regime of DLVO interaction. On a cleaved calcite surface, the positive >Ca^2+^ sites generate repulsion, whereas the negative >CO_3_^2−^ sites induce specific attraction. The two forces are neutralized in DIW, as shown in Figure 9a. The incorporation of positive PDIs (Ca^2+^ and Mg^2+^) screens the negative >CO_3_^2−^ sites and increases the repulsive force, thus enhancing the paste flow (see Figure 9b). On the contrary, the negative PDIs screen the positive >Ca^2+^ sites and promote the interparticle attraction (Figure 9c), which inhibits the suspension flow. Based on this model, it is expected that the effect of positive PDIs follows the DLVO model, i.e., the moderate adsorption of Ca^2+^ and Mg^2+^ enhances the EDL repulsion, and the negative PDIs act adversely even though the calcite is highly negatively charged. As for indifferent ions such as Na^+^, K^+^ and NO_3_^−^, an increasing concentration will change the ionic strength and the Debye length, considering that the initial charge of calcite is weak, so they have negligible effect on calcite paste rheology, which also follows the DLVO model.

The secondary difference between Ca^2+^ and Mg^2+^ on calcite paste rheology is ascribed to different covalency indices and affinity to the calcite surface, where weakly attracted Mg^2+^ expands the Helmholtz layer and Debye length, and in turn enhances the EDL repulsion. As for negative PDIs, the OH^−^ binds more >Ca^2+^ sites, and the deprotonation process releases more >CO_3_^2−^ sites, thus producing a stronger specific attraction and leading to a more rigid paste. In contrast, the weakly adsorbed SO_4_^2−^ cannot entirely cover all >Ca^2+^ sites and produces the smallest specific attraction.

## 5. Conclusions

In this work, we systematically investigated the influence of positive PDIs (Ca^2+^ and Mg^2+^), negative PDIs (OH^−^, CO_3_^2−^ and SO_4_^2−^) and indifferent ions (Na^+^ and K^+^) on the zeta potential and rheological behavior of calcite paste. Zeta potential evolution and the ionic adsorption model were illustrated with different EDL models, and the rheological behavior of calcite paste was analyzed by interparticle force. Throughout this paper, the following conclusions can be drawn:

(1) The adsorption of positive PDIs elevates the positive charge of calcite, whereas the negative PDIs make the calcite more negatively charged. Calcite exhibits higher zeta potential in Ca^2+^ than Mg^2+^, and in negative PDI solutions follows the order of $\left|\xi_{CO_{3}^{2-}}\right|>\left|\xi_{SO_{4}^{2-}}\right|>\left|\xi_{OH^{-}}\right|$. Indifferent ions such as Na^+^, K^+^ and NO_3_^2−^ slightly change the zeta potential by tuning the ionic strength and Debye length.
(2) The natural calcite is constantly more negative than synthetic calcite due to the existence of impurities, indicating that the detection of an IEP by zeta potential test is insufficient for PDI identification. Fajans’s rule, denoting the correlation between ionic adsorption and solubility of precipitates, provides a practical method for PDI identification. In addition, the rheology test also indicates a feasible approach, since the calcite paste rheology is insensitive to indifferent ions such as Na^+^, K^+^ and NO_3_^−^.
(3) The incorporation of positive PDIs significantly increases the positive charge of calcite and enhances the suspension flow, which basically follows the DLVO model. Ca^2+^ has a higher capability of improving calcite charge than Mg^2+^, whereas the Mg^2+^ is more capable in improving the suspension flow. The secondary differences may result from a different covalency index and affinity to the calcite surface, where Ca^2+^ is more strongly bound by calcite and the Mg^2+^ bond is relatively weak.
(4) The increasing negative PDIs make the calcite negatively charged leading to a more viscous paste. Calcite paste exhibits non-DLVO behavior with negative PDI (OH^−^, CO_3_^2−^ and SO_4_^2−^) solutions, where negatively charged calcite paste exhibits much higher viscosity. Specific attraction and lattice site screening induced by negative PDIs may be the reason for this phenomenon. The deprotonation reaction by OH^−^ generates the highest specific attraction in calcite paste, thus inducing the greatest rigidity and viscosity, while the weakly attracted SO_4_^2−^ induces a much smaller attraction. The interaction model, denoting an extended DLVO theory, provides a rational explanation for the non-DLVO behavior.

This study revealed how ionic admixtures affect the kinetics of calcite paste, and the results are inspiring for tuning the workability of carbonate suspensions via surface charge control. Relative mechanisms have broad applications in many industrial fields such as paper manufacturing, building materials and heritage conservation.

## Figures and Tables

**Table 1:** The chemical composition of natural and synthetic calcites (wt.%).

	CaCO_3_	MgCO_3_	SiO_2_	Al_2_O_3_	Fe_2_O_3_	SO_3_	K_2_O	SrO	NiO	MnO	Cr_2_O_3_
Syntheticcalcite	>99	0.05	-	-	0.001	0.01	0.005	0.05	-	-	-
Natural calcite	91.05	7.19	1.21	0.30	0.11	0.05	0.03	0.02	0.02	0.01	0.01

**Table 2:** The pH of NaOH and Na_2_CO_3_ solution at different concentrations.

pH	1 mM	10 mM	20 mM	40 mM
NaOH	10.8	12.0	12.3	12.6
Na_2_CO_3_	10.3	11.1	11.3	11.4

## Data Availability

The original contributions presented in this study are included in the article. Further inquiries can be directed to the corresponding author.

## References

[1] Hofmann S., Voïtchovsky K., Spijker P., Schmidt M., Stumpf T. (2016). Visualising the molecular alteration of the calcite (104)—Water interface by sodium nitrate. Sci. Rep..

[2] Knauss K.G., Johnson J.W., Steefel C.I. (2005). Evaluation of the impact of CO_2_, co-contaminant gas, aqueous fluid and reservoir rock interactions on the geologic sequestration of CO2. Chem. Geol..

[3] Guo H., Kovscek A.R. (2019). Investigation of the effects of ions on short-range non-DLVO forces at the calcite/brine interface and implications for low salinity oil-recovery processes. J. Colloid Interface Sci..

[4] Sadatshojaei E., Jamialahmadi M., Esmaeilzadeh F., Wood D.A., Ghazanfari M.H. (2019). The impacts of silica nanoparticles coupled with low-salinity water on wettability and interfacial tension: Experiments on a carbonate core. J. Dispers. Sci. Technol..

[5] Heberling F., Bosbach D., Eckhardt J.-D., Fischer U., Glowacky J., Haist M., Kramar U., Loos S., Müller H.S., Neumann T. (2014). Reactivity of the calcite–water-interface, from molecular scale processes to geochemical engineering. Appl. Geochem..

[6] Papo A., Piani L. (2005). Rheological Behavior of Calcite Slurries: Effect of Deflocculant Addition. Part. Sci. Technol..

[7] Liberto T., Le Merrer M., Barentin C., Bellotto M., Colombani J. (2017). Elasticity and yielding of a calcite paste: Scaling laws in a dense colloidal suspension. Soft Matter.

[8] Liberto T., Barentin C., Colombani J., Costa A., Gardini D., Bellotto M., Le Merrer M. (2019). Simple ions control the elasticity of calcite gels via interparticle forces. J. Colloid Interface Sci..

[9] Benachour Y., Davy C.A., Skoczylas F., Houari H. (2008). Effect of a high calcite filler addition upon microstructural, mechanical, shrinkage and transport properties of a mortar. Cem. Concr. Res..

[10] Pourchet S., Pochard I., Brunel F., Perrey D. (2013). Chemistry of the calcite/water interface: Influence of sulfate ions and consequences in terms of cohesion forces. Cem. Concr. Res..

[11] Goergens J., Manninger T., Goetz-Neunhoeffer F.J.C., Research C. (2020). In-situ XRD study of the temperature-dependent early hydration of calcium aluminate cement in a mix with calcite. Cem. Concr. Res..

[12] Gulmez N. (2020). Roles of aluminium shavings and calcite on engineering properties of cement-based composites. J. Clean. Prod..

[13] Mikanovic N., Jolicoeur C. (2008). Influence of superplasticizers on the rheology and stability of limestone and cement pastes. Cem. Concr. Res..

[14] Deraguin B., Landau L. (1941). Theory of the stability of strongly charged lyophobic sols and of the adhesion of strongly charged particles in solution of electrolytes. Prog. Surf. Sci..

[15] Verwey E.J.W., Overbeek J.T.G., Van Nes K. (1948). Theory of the Stability of Lyophobic Colloids: The Interaction of Sol Particles Having an Electric Double Layer.

[16] Thompson D.W., Pownall P.G. (1989). Surface electrical properties of calcite. J. Colloid Interface Sci..

[17] Cicerone D.S., Regazzoni A.E., Blesa M.A. (1992). Electrokinetic properties of the calcite/water interface in the presence of magnesium and organic matter. J. Colloid Interface Sci..

[18] Nyström R., Lindén M., Rosenholm J.B. (2001). The Influence of Na^+^, Ca^2+^, Ba^2+^, and La^3+^ on the ζ Potential and the Yield Stress of Calcite Dispersions. J. Colloid Interface Sci..

[19] Pierre A., Lamarche J.M., Mercier R., Foissy A., Persello J. (1990). Calcium as Potential Determining Ion in Aqueous Calcite Suspensions. J. Dispers. Sci. Technol..

[20] Huang Y.C., Fowkes F.M., Lloyd T.B., Sanders N.D. (1991). Adsorption of calcium ions from calcium chloride solutions onto calcium carbonate particles. Langmuir.

[21] Eriksson R., Merta J., Rosenholm J.B. (2007). The calcite/water interface: I. Surface charge in indifferent electrolyte media and the influence of low-molecular-weight polyelectrolyte. J. Colloid Interface Sci..

[22] Moulin P., Roques H. (2003). Zeta potential measurement of calcium carbonate. J. Colloid Interface Sci..

[23] Foxall T., Peterson G.C., Rendall H.M., Smith A.L. (1979). Charge determination at calcium salt/aqueous solution interface. J. Chem. Soc. Faraday Trans. 1 Phys. Chem. Condens. Phases.

[24] Douglas H.W., Walker R.A. (1950). The electrokinetic behaviour of iceland spar against aqueous electrolyte solutions. Trans. Faraday Soc..

[25] Song J., Zeng Y., Wang L., Duan X., Puerto M., Chapman W.G., Biswal S.L., Hirasaki G.J. (2017). Surface complexation modeling of calcite zeta potential measurements in brines with mixed potential determining ions (Ca^2+^, CO_3_^2−^, Mg^2+^, SO_4_^2−^) for characterizing carbonate wettability. J. Colloid Interface Sci..

[26] Van Cappellen P., Charlet L., Stumm W., Wersin P. (1993). A surface complexation model of the carbonate mineral-aqueous solution interface. Geochim. Cosmochim. Acta.

[27] Heberling F., Trainor T.P., Lutzenkirchen J., Eng P., Denecke M.A., Bosbach D. (2011). Structure and reactivity of the calcite-water interface. J. Colloid Interface Sci..

[28] Pokrovsky O.S., Schott J. (2002). Surface chemistry and dissolution kinetics of divalent metal carbonates. Environ. Sci. Technol..

[29] Alroudhan A., Vinogradov J., Jackson M.D. (2016). Zeta potential of intact natural limestone: Impact of potential-determining ions Ca, Mg and SO_4_. Colloids Surf. A Physicochem. Eng. Asp..

[30] Smallwood P.V. (1977). Some aspects of the surface chemistry of calcite and aragonite Part I: An electrokinetic study. Colloid Polym. Sci..

[31] Zhang P., Austad T. (2006). Wettability and oil recovery from carbonates: Effects of temperature and potential determining ions. Colloids Surf. A Physicochem. Eng. Asp..

[32] Israelachvili J.N. (2011). Intermolecular and Surface Forces.

[33] Røyne A., Dalby K.N., Hassenkam T. (2015). Repulsive hydration forces between calcite surfaces and their effect on the brittle strength of calcite-bearing rocks. Geophys. Res. Lett..

[34] Diao Y., Espinosa-Marzal R.M. (2016). Molecular insight into the nanoconfined calcite-solution interface. Proc. Natl. Acad. Sci. USA.

[35] Grasso D., Subramaniam K., Butkus M., Strevett K., Bergendahl J. (2002). A review of non-DLVO interactions in environmental colloidal systems. Rev. Environ. Sci. Bio/Technol..

[36] Javadi S., Røyne A. (2018). Adhesive forces between two cleaved calcite surfaces in NaCl solutions: The importance of ionic strength and normal loading. J. Colloid Interface Sci..

[37] Marchuk A., Rengasamy P. (2011). Clay behaviour in suspension is related to the ionicity of clay–cation bonds. Appl. Clay Sci..

[38] Huang J., Xu W., Chen H., Xu G. (2021). Elucidating how ionic adsorption controls the rheological behavior of quartz and cement-quartz paste. Constr. Build. Mater..

[39] Zhu Y., Ali A., Dang A., Wandel A.P., Bennett J.M. (2019). Re-examining the flocculating power of sodium, potassium, magnesium and calcium for a broad range of soils. Geoderma.

[40] Allen J.B., Larry R.F. (2001). Electrochemical Methods Fundamentals and Applications.

[41] Wolthers M., Charlet L., Van Cappellen P. (2008). The surface chemistry of divalent metal carbonate minerals; a critical assessment of surface charge and potential data using the charge distribution multi-site ion complexation model. Am. J. Sci..

[42] Al Mahrouqi D., Vinogradov J., Jackson M.D. (2017). Zeta potential of artificial and natural calcite in aqueous solution. Adv. Colloid Interface Sci..

[43] Kasha A., Al-Hashim H., Abdallah W., Taherian R., Sauerer B. (2015). Effect of Ca^2+^, Mg^2+^ and SO_4_^2−^ ions on the zeta potential of calcite and dolomite particles aged with stearic acid. Colloids Surf. A Physicochem. Eng. Asp..

[44] Sondi I., Bišćan J., Vdović N., Škapin S.D.J.C. (2009). The electrokinetic properties of carbonates in aqueous media revisited. Colloids Surfaces A Physicochem. Eng. Asp..

[45] Mahani H., Keya A.L., Berg S., Nasralla R. (2018). Electrokinetics of carbonate/brine interface in low-salinity waterflooding: Effect of brine salinity, composition, rock type, and pH on?-potential and a surface-complexation model. SPE J..

[46] Chen L., Zhang G., Wang L., Wu W., Ge J.J.C., Physicochemical S.A., Aspects E. (2014). Zeta potential of limestone in a large range of salinity. Colloids Surfaces A Physicochem. Eng. Asp..

[47] Vdović N., Bišćan J.J.C., Physicochemical S.A., Aspects E. (1998). Electrokinetics of natural and synthetic calcite suspensions. Colloids Surf. A Physicochem. Eng. Asp..

[48] Vdović N. (2001). Electrokinetic behaviour of calcite—The relationship with other calcite properties. Chem. Geol..

[49] Zhang Y., Dawe R. (2000). Influence of Mg^2+^ on the kinetics of calcite precipitation and calcite crystal morphology. Chem. Geol..

[50] Zarga Y., Boubaker H.B., Ghaffour N., Elfil H. (2013). Study of calcium carbonate and sulfate co-precipitation. Chem. Eng. Sci..

[51] Curti E. (1999). Coprecipitation of radionuclides with calcite: Estimation of partition coefficients based on a review of laboratory investigations and geochemical data. Appl. Geochem..

[52] Kolthoff I.M., MacNevin W.M. (2002). The Adsorption of Barium Salts on Barium Sulfate from Solutions in 50% Ethanol. J. Am. Chem. Soc..

[53] Stone H.E.N. (2013). Valency relations between alkali and alkali earth elements and elements of second and third long periods. Mater. Sci. Technol..

